# Age-Related Changes in Synaptic Plasticity Associated with Mossy Fiber Terminal Integration during Adult Neurogenesis

**DOI:** 10.1523/ENEURO.0030-20.2020

**Published:** 2020-05-20

**Authors:** Karl D. Murray, Xiao-Bo Liu, Anna N. King, Julie D. Luu, Hwai-Jong Cheng

**Affiliations:** 1 Center for Neuroscience; 2 Department of Psychiatry and Behavioral Neuroscience; 3 Department of Neurobiology, Physiology and behavior; 4 Department of Pathology and Laboratory Medicine, University of California, Davis, Davis, CA 95618

**Keywords:** aging, conditional transgenic, giant synapse, stratum lucidum, synaptogenesis

## Abstract

Mouse hippocampus retains the capacity for neurogenesis throughout lifetime, but such plasticity decreases with age. Adult hippocampal neurogenesis (AHN) involves the birth, maturation, and synaptic integration of newborn granule cells (GCs) into preexisting hippocampal circuitry. While functional integration onto adult-born GCs has been extensively studied, maturation of efferent projections onto CA3 pyramidal cells is less understood, particularly in aged brain. Here, using combined light and reconstructive electron microscopy (EM), we describe the maturation of mossy fiber bouton (MFB) connectivity with CA3 pyramidal cells in young adult and aged mouse brain. We found mature synaptic contacts of newborn GCs were formed in both young and aged brains. However, the dynamics of their spatiotemporal development and the cellular process by which these cells functionally integrated over time were different. In young brain newborn GCs either formed independent nascent MFB synaptic contacts or replaced preexisting MFBs, but these contacts were pruned over time to a mature state. In aged brain only replacement of preexisting MFBs was observed and new contacts were without evidence of pruning. These data illustrate that functional synaptic integration of AHN occurs in young adult and aged brain, but with distinct dynamics. They suggest elimination of preexisting connectivity is required for the integration of adult-born GCs in aged brain.

## Significance Statement

Most neurons in the brain are generated early in development then maintained in adulthood. However, in dentate gyrus, granule cells (GCs) continue to be generated throughout life. Adult-born GCs are important for certain forms of learning and memory, but how these neurons integrate into preexisting hippocampal circuitry is not well known. In addition, whether integration in aged brain, where cognitive function is reduced, is different from young adult brain has not been tested. Here, we show that in young adult brains newly generated GCs integrate synaptic outputs by forming *de novo* synaptic contacts as well as taking over preexisting ones. By contrast, in aged brain only synaptic replacement is observed. These observations could be relevant to cognitive decline in aging.

## Introduction

Certain discrete regions of the adult brain retain the capacity for continued neurogenesis throughout life. This unique form of neurodevelopment allows those areas to continue exhibiting neuronal plasticity into adulthood. Adult neurogenesis uniquely involves integration of new neurons into a functional neuronal circuit, which requires proper development of synaptic inputs onto their dendrites and synaptic outputs from their axons. In contrast to early developmental neurogenesis, adult-born neurons must integrate into a fully developed neuronal circuit, but how the integration process is regulated is largely unknown. Adult neurogenesis has been implicated in various human disorders such as Alzheimer’s disease, depression, and drug addiction (Sahay and Hen, 2007; Noonan et al., 2010). Understanding the underlying mechanisms regulating adult neurogenesis might provide some insights into the causes of these diseases.

In adult hippocampal neurogenesis (AHN), hippocampal dentate gyrus granule cells (GCs) receive major synaptic inputs from local interneurons and perforant pathway axons originating in entorhinal cortex, and send mossy fibers through the hilus and into stratum lucidum of CA3 (CA3sl), where large mossy fiber boutons (MFBs) form en passant synaptic contacts with complex dendritic protrusions termed thorny excrescences (TEs) on dendrites of CA3 pyramidal neurons (Toni et al., 2007, 2008; Toni and Sultan, 2011; Song et al., 2012; Braun and Jessberger, 2014; Vadodaria and Jessberger, 2014; Kempermann et al., 2015). In mice, GCs are continuously generated throughout adult life. Recent evidence has demonstrated that neurogenesis in adult hippocampus recapitulates aspects of embryonic development albeit on a more protracted timeline (Espósito et al., 2005; Overstreet-Wadiche et al., 2006; Zhao et al., 2006; Duan et al., 2007; Faulkner et al., 2008). It has been suggested that newly formed synapses onto adult-born GCs replace preexisting contacts thereby maintaining overall synaptic numbers (Toni et al., 2007, 2008).

Considerable research efforts have focused on development and integration of newborn GCs in young adult hippocampus [up to three months (3 M)]. However, virtually nothing is known regarding neurogenesis in the aged hippocampus. Although neurogenesis is significantly reduced in aged brain, GCs generated in 10-M-old mice still exhibit the ability to differentiate into a morphologically mature neuron (Morgenstern et al., 2008; Ahlenius et al., 2009). Studies in young adult mice indicate that adult-born GCs only account for as little as 14% of the mature GC layer (GCL), and this number is, surprisingly, not increased by potent physiological stimulation (Ninkovic and Götz, 2007; Ninkovic et al., 2007). Ultimately, the contribution of adult-born neurons to the function of hippocampal circuits is determined by those neurons which are integrated into the existing network and not by the number of neurons that are generated. Therefore, it is essential to understand the integration of adult-born neurons into a mature circuit to understand how neurogenesis influences function.

Here, we investigate the maturation and insertion of newborn hippocampal GCs in young adult mice and determine whether similar patterns of development occur in aged brain. Using a conditional transgenic reporter mouse line, we efficiently label neuronal progenitors and their progeny, in young adult and importantly, aged brain, and perform a quantitative analysis of morphometric changes to newborn MFBs as they integrate into existing hippocampal circuits. In young adult mice newborn MFBs mature over the course of 16 weeks displaying a large reduction in number and concomitant increases in bouton size. Ultrastructural analysis at electron microscopic (EM) level indicates these changes are reflecting *de novo* synaptogenesis as well as replacement of existing terminals. Similar synaptic plasticity was not observed in aged brain. In aged brain, bouton number increased over time while terminal size decreased. At the ultrastructural level *de novo* synapse formation was not observed but rather a synapse replacement strategy was exclusively employed. These results suggest the behavioral impact of adult neurogenesis in aged brain may be different to that in young adult.

## Materials and Methods

### Transgenic *Gli1-CreER^T2^* mice

**Figure 1.**
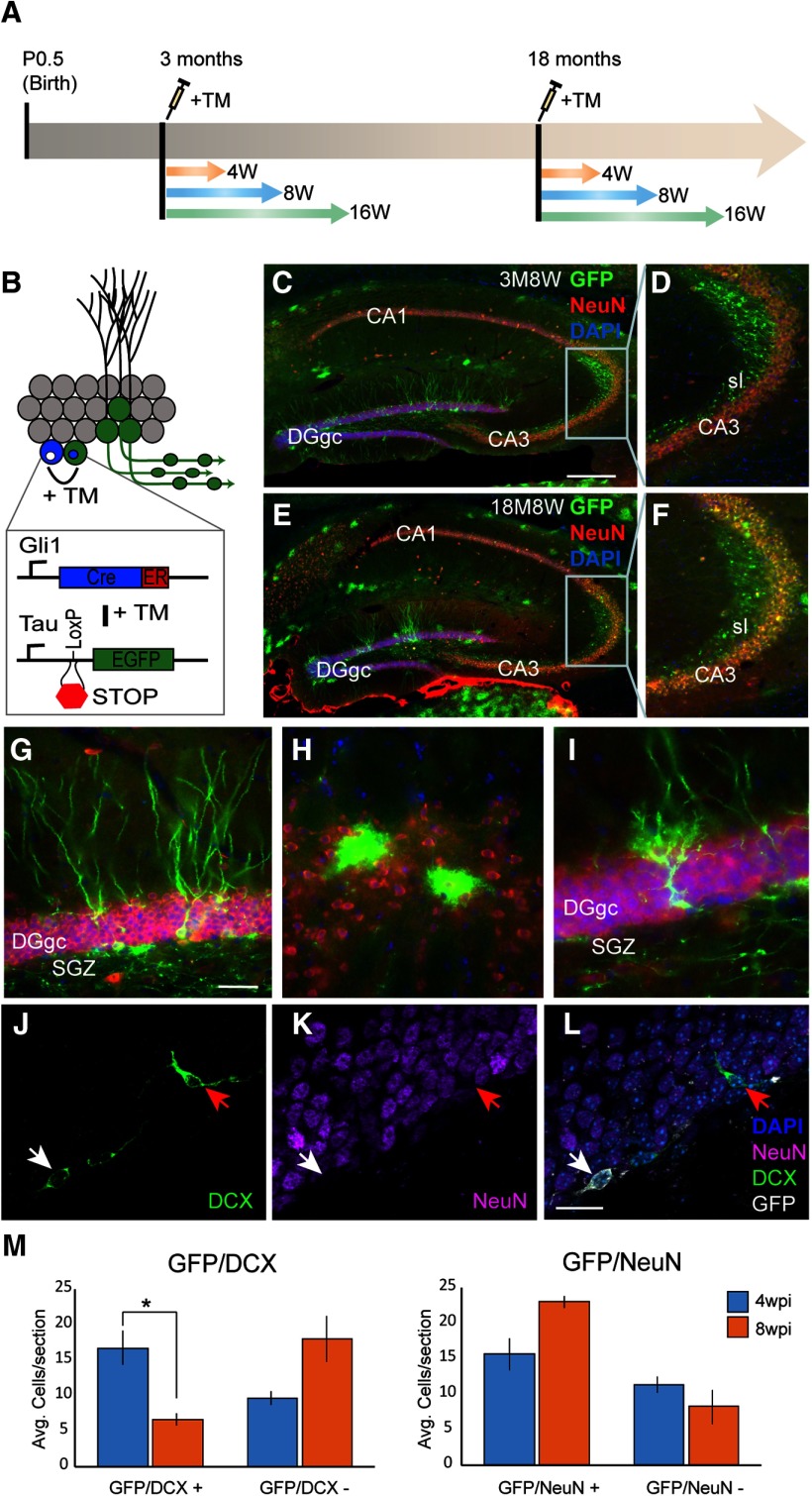
GliCreGFP transgenic mice enable conditional birth-dating and morphometric analysis of newborn hippocampal GCs in adult and aged animals. ***A***, Strategy for newborn GC birth dating. Administration of TM at different ages turns on GFP expression in progenitors and enables tracking of subsequent daughter GCs at various time points (e.g., 4, 8, or 16 weeks) postinjection. ***B***, Schematic illustration of conditional GFP expression strategy in *Gli1* responsive progenitors post-TM administration. ***C***, Epifluorescent images of immunofluorescent labeling for GFP and NeuN in a 3 M adult hippocampus 8W post-TM administration. ***D***, Higher magnification image taken from boxed region in ***C***. ***E***, Epifluorescent images of immunofluorescent labeling for GFP and NeuN in an 18 M-aged hippocampus 8W post-TM administration. ***F***, Higher magnification image taken from boxed region in ***E***. ***G***, Morphologically mature looking dentate gyrus GCs immunolabeled for GFP 8W post-TM. ***H***, Astrocyte glia in subiculum immunolabeled for GFP 8 wpi. ***I***, Radial glial-like progenitors immunolabeled for GFP are observed in the dentate gyrus bordering the subgranular zone 8W post-TM. ***J–L***, Cell-type marker analysis of GFP-labeled cells by immunolabeling. At 4W post-TM, some GFP cells expressed the immature neuronal marker DCX but not mature neuronal marker NeuN (white arrow). Not all DCX cells were GFP-positive (red arrow). ***M***, Cell counts of DCX/NeuN/GFP cells shows a progressive decrease in number of DCX/GFP cells and increase in NeuN/GFP cells from 4W to 8W post-TM (GFP/DCX+: 4 wpi: *n* = 9, 8 wpi: *n* = 9, *p* < 0.05, Student’s *t* test). DGgc, dentate gyrus GC; CA1-3, cornu ammonis region1-3; SGZ, subgranular zone; sl, stratum lucidum; sr, stratum radiatum. Scale bars = 1 mm (***C–F***), 100 μm (***G–I***), 50 μm (***J–L***); *p < 0.05.

**Figure 2.**
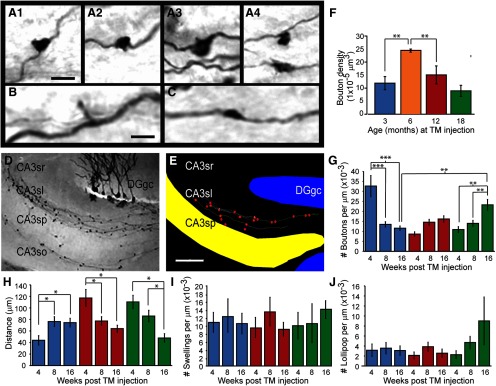
Spatiotemporal maturation of adult-born GC mossy fiber axon terminals in adult and aged mice. ***A***, Examples of large complex MFBs labeled by immunohistochemistry with an antibody against GFP. ***B***, Example of a lollipop bouton. Note short thin collateral and small bulbous terminal. ***C***, Example of an immunolabeled en passant swelling in newborn mossy fiber axon. Note the small diameter of the swelling and that it is in line with the axon. ***D***, Low-power image of immunolabeled adult-born mossy fibers and boutons in region CA3. ***E***, Neurolucida reconstructions of adult-born mossy fibers and boutons taken from image in ***D***. ***F***, Spatial density of labeled MFBs in stratum lucidum at 8W post-TM administration in adult (3 M; *n* = 400), midlife (6 M; *n* = 252), and aged (12 M and 15 M; *n* = 249 and *n* = 261, respectively) animals. ***G***, Axonal density measures of MFBs at various times post-TM administration in adult and aged animals (3 M: *n* = 135, 12 M: *n* = 146, 18 M: *n* = 128). ***H***, Interbouton spacing measures of MFBs at various times post-TM administration in adult and aged animals (3 M: *n* = 135, 12 M: *n* = 146, 18 M: *n* = 128). ***I***, Axonal density measures of en passant swellings at various times post-TM administration in adult and aged animals (3 M: *n* = 110, 12 M: *n* = 116, 18 M: *n* = 90). ***J***, Axonal density measures of mossy fiber lollipop boutons at various times post-TM administration in adult and aged animals (3 M: *n* = 25, 12 M: *n* = 30, 18 M: *n* = 38). CA3, cornu ammons region 3; DGgc, dentate gyrus GCL; sr, stratum radiatum; sl, stratum lucidum; sp, stratum pyramidale; so, stratum oriens. Scale bars = 5 μm (***A***), 10 μm (***B***, ***C***), 500 μm (***E***); *p < 0.05, **p < 0.01, ***p < 0.005, two-way ANOVA followed by Student’s *t* test. All values are mean ± SEM.

**Figure 3.**
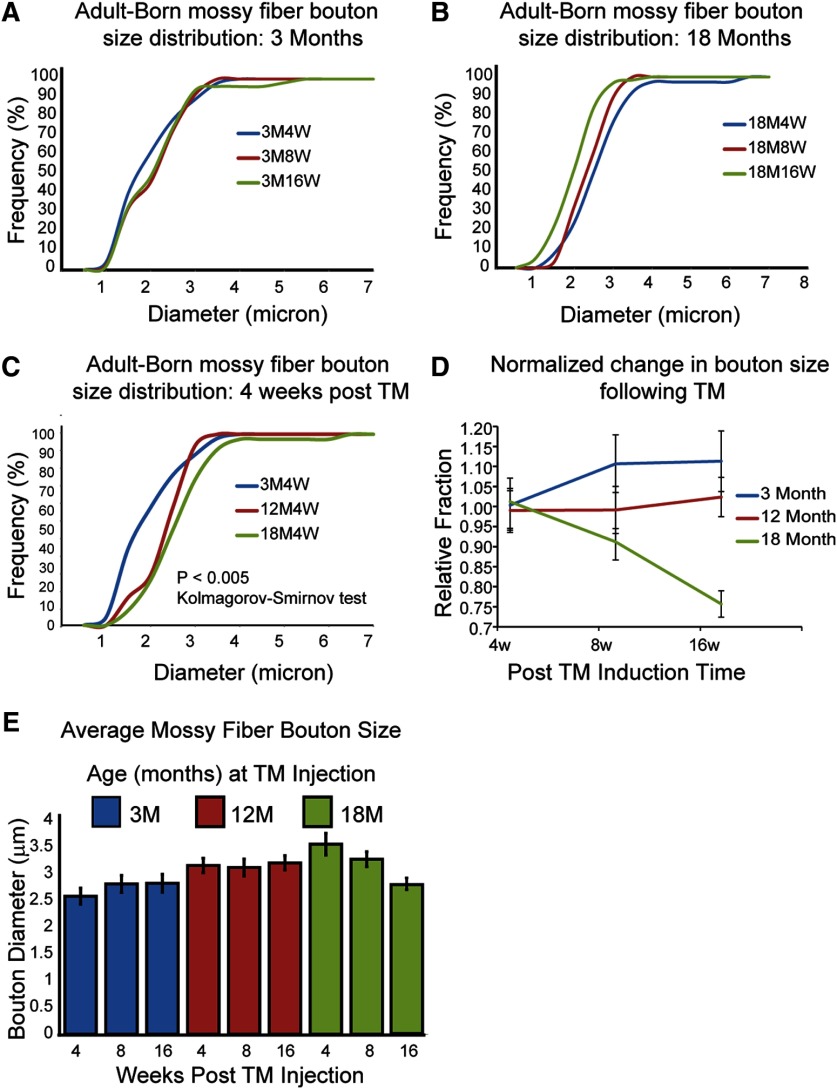
Opposing developmental trajectories in MFB size during adult and aged hippocampal neurogenesis. ***A***, Newborn MFB size distributions in 3 M adult hippocampus, 4W, 8W, and 16W post-TM administration (4 wpi; *n* = 47, 8 wpi: *n* = 37, 16 wpi: *n* = 51). ***B***, Newborn MFB size distribution in 18 M-aged hippocampus, 4W, 8W, and 16W post-TM administration (4 wpi: *n* = 38, 8 wpi: *n* = 28, 16 wpi: *n* = 62). ***C***, Comparison of the relative newborn MFB size distribution 4W post-TM administration in 3-M-, 12-M-, and 18-M-old animals. Note the significantly smaller size distribution in 3 M animals relative to 12 M and 18 M animals (*p* < 0.005 Kolmogorov–Smirnov test). ***D***, Developmental changes in MFB size occurred in opposite directions between 3 M and 18 M brain. Changes in average size plotted relative to 4W post-TM show increases in MFB size in 3 M but decreases in 18 M animals. Average MFB size remained relatively unchanged in 12 M animals. ***E***, Averaged MFB sizes. The size is determined by measuring the diameter of each MFB. Values in ***D***, ***E*** are mean ± SEM.

**Figure 4.**
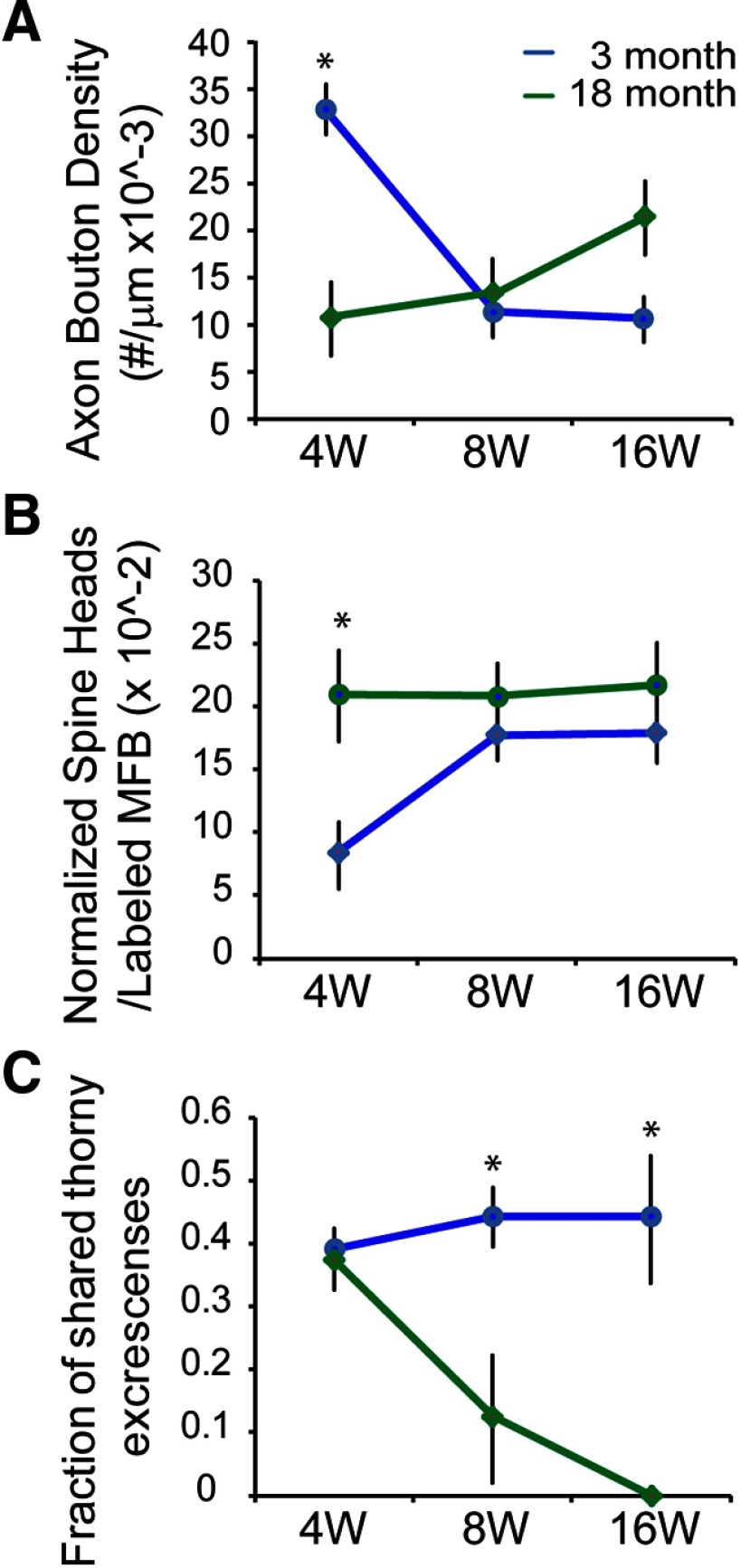
Quantitative analysis of serial EM reconstructed adult-born MFB onto postsynaptic TEs. ***A***, Axonal bouton density (*n* = 50–100 boutons per time point; 3M4 wpi: *n* = 63, 18M4 wpi, *n* = 78, *p* < 0.05). ***B***, Normalized average number of TE spine heads in the GFP-labeled newborn MFB (*n* = 3–4 reconstructed MFBs per time point; 3M4 wpi: *n* = 4, 18M4 wpi: *n* = 3). ***C***, Percentage of TEs shared by GFP-labeled and non-labeled MFBs were analyzed in adult (3 M) and aged (18 M) hippocampus, 4W, 8W, and 16W post-TM administration (*n* = 8–15 reconstructed TEs per time point). *p < 0.05, Student’s *t* test. Values are mean ± SEM (***A***, ***B***) or ±SD (***C***).

**Figure 5.**
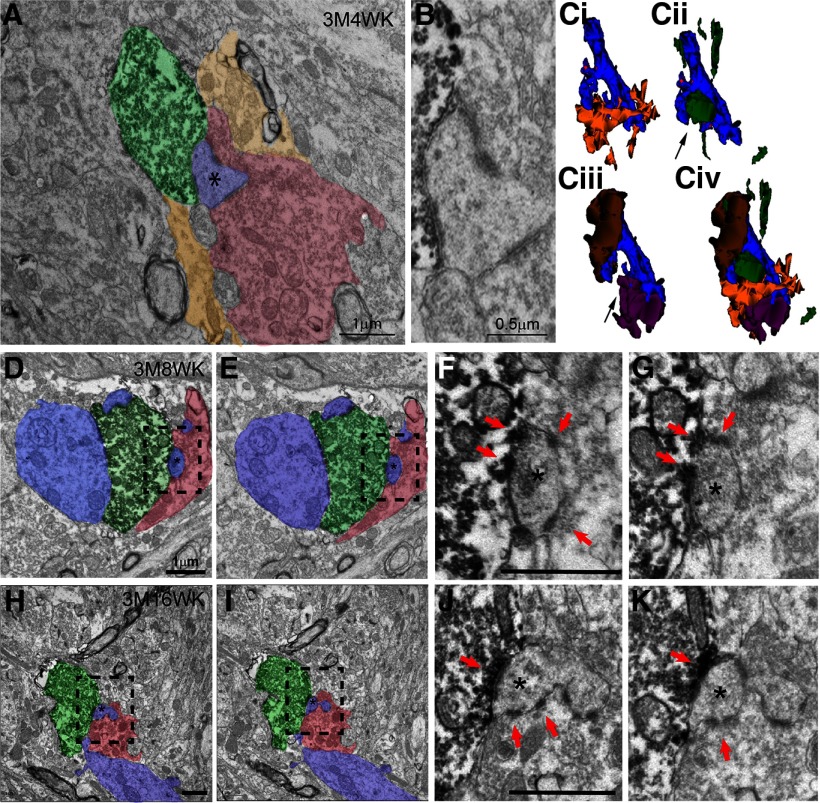
Electron micrographs and 3D reconstructions demonstrating sharing of postsynaptic TEs by newborn and mature MFBs in 3 M adult mice at different times post-TM injection. ***A–C***, Representative 3D reconstruction of a 3M4W newborn MFB. Shown in ***A*** is an electron micrograph of GFP-labeled newborn MFB (green) at 3M4W and non-GFP-labeled mature MFB (red) both forming asymmetrical synaptic contacts with the same spine head (blue, asterisk). Surrounding glia processes are also marked (yellow). Note the glia processes are not seen in the proximity to the postsynaptic density and synaptic cleft. The synaptic contact region and the shared spine head are magnified and shown in ***B***. Different combinations of 3D reconstruction of this 3M4W newborn MFB with postsynaptic dendrite, neighboring mature MFBs and associated glial cell are shown in ***Ci–Civ***. These include: (***Ci***) postsynaptic dendrite (blue) giving rise to two TEs and associated glial processes (yellow), (***Cii***) GFP-labeled newborn mossy fiber and its bouton (green) forming synaptic contact with the spine head of the smaller TE (arrow), (***Ciii***) two non-GFP-labeled mature MFBs (brown and purple) forming contacts with the two TEs, respectively, and (***Civ***) a combination of all components. Note the small TE (arrow) is shared by the newborn MFB (green) and mature MFB (purple) as seen in the electron micrograph in ***A***. ***D–G***, Representative electron micrographs of a 3M8W newborn MFB. Shown in ***D***, ***E*** are two serial EM sections of a GFP-labeled MFB (green) at 3M8W shares a postsynaptic spine head (blue, asterisk) with a non-GFP-labeled mature MFB (red). Higher magnification boxed regions in ***D***, ***E*** are shown in ***F***, ***G***, respectively. The asymmetric synaptic contacts are marked with red arrowheads. ***H–K***, Representative electron micrographs of a 3M16W newborn MFB. Shown in ***H***, ***I*** are two serial EM sections of a GFP-labeled MFB (green) at 3M16W shares a postsynaptic spine head (blue, asterisk) with a non-GFP-labeled mature MFB (red). Higher magnification boxed regions in ***H***, ***I*** are shown in ***J***, ***K***, respectively. The asymmetric synaptic contacts are marked with red arrowheads. Scale bars = 1 μm.

**Figure 6.**
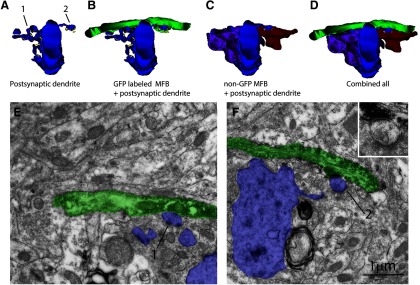
Smaller newborn MFB at 3M4W can share a postsynaptic spine head with mature MFB. ***A–D***, 3D reconstruction of a newborn mossy fiber at 3M4W forming contacts with postsynaptic dendrite of a CA3 pyramidal cell. The 3M4W GFP-labeled mossy fiber (green) forms two small MFB contact sites, labeled as 1 and 2, with TEs on the dendrite of a CA3 pyramidal cell (blue). The two TE also form contacts with mature MFBs at site 1 (purple) and site 2 (red). ***E***, Electron micrograph shows that MFB at site 1 (green) does not form asymmetric synaptic contact with TE (blue), which in turn forms multiple asymmetric synaptic contacts with the non-GFP-labeled mature MFB. ***F***, Electron micrograph shows that MFB at site 2 (green) forms an asymmetrical synaptic contact with a spine head of TE (blue) that is also contacted by another non-GFP-labeled mature MFB. Inset shows a high magnification of the shared spine head and the asymmetrical synaptic contacts. Scale bar = 1 μm.

**Figure 7.**
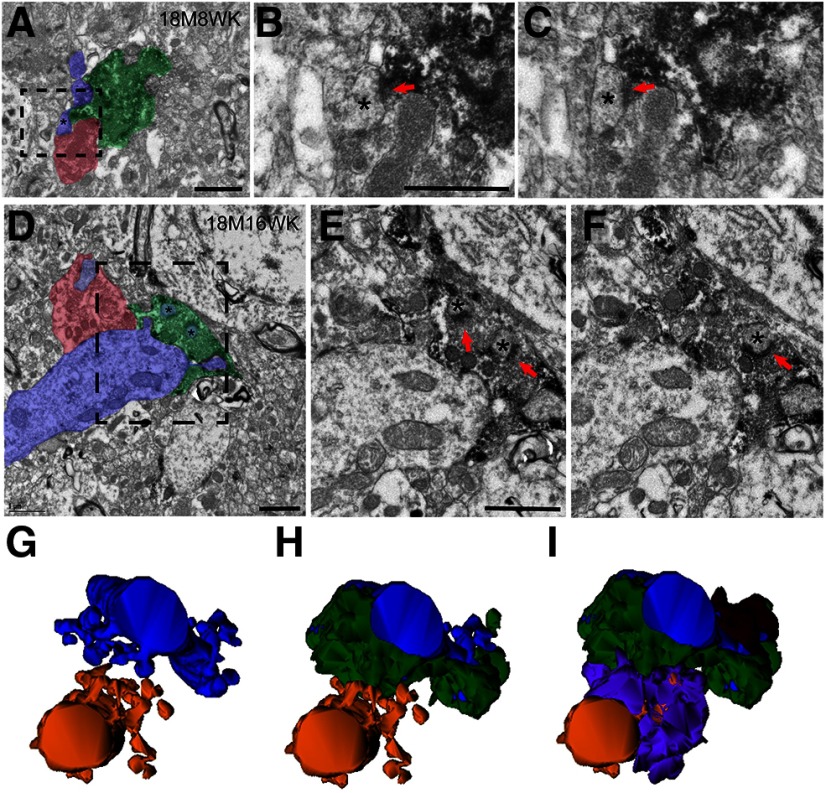
Electron micrographs and 3D reconstructions demonstrating sharing of postsynaptic TEs by newborn and mature MFBs in 18 M adult mice at 8W post-TM but not at 16W post-TM. ***A–C***, Representative electron micrograph shows an 18M8W GFP-labeled MFB (green) shares a small postsynaptic spine head (blue, asterisk) with a non-GFP-labeled mature MFB (red). Serial micrographs of the boxed area in ***A*** are magnified and shown in ***B***, ***C***. Note the labeled newborn MFB and non-labeled mature MFB are very close to each other, but the axoplasm membranes clearly separate two boutons, the serial section analysis also show that the two postsynaptic densities are separated. Red arrowheads mark the asymmetric synaptic contacts by the newborn MFB. ***D–F***, Representative electron micrographs of a 18M16W newborn MFB. A GFP-labeled 18M16W newborn MFB (green) and a non-GFP-labeled mature MFB (red) form asymmetric synaptic contacts with spine heads derived from different TEs (blue). They do not share contacts with spine heads (asterisk). Serial micrographs of the boxed area in ***D*** are magnified and shown in ***E***, ***F***. Red arrowheads mark the asymmetric synaptic contacts by the newborn MFB. ***G–I***, 3D reconstruction of a 18M16W newborn MFB that does not show sharing. The two reconstructed postsynaptic dendrites (blue and yellow) have a total of three TEs (***G***). The GFP-labeled MFB (green) forms synaptic contacts with one TE from the top dendrite (blue; ***H***). This MFB does not form synaptic contact with spine heads from other TEs. Combined image (***I***) show that the other two TEs are contacted by two non-GFP-labeled MFBs (brown and purple). Again, these two mature MFBs confine their synaptic contacts to only the TE they cover; no sharing is observed among them. Scale bars = 1 μm.

All procedures involving mice were approved by the University Institutional Animal Care and Use Committee and were performed in strict accordance with the Guide for the Care and Use of Laboratory Animals of the NIH. Twelve mice were used to perform cell counts using cell-type markers (Fig. 1). A total of 32 animals were used for axon MFB morphometric reconstructions and analysis (Figs. 2, 3) and an additional 18 animals were used for EM (Figs. 4-7). Mice were maintained under standard light-dark cycles and allowed to feed and drink *ad libitum*. Mixed number of male and female mice were used in this study.

Transgenic *Gli1-CreER^T2^* mice were kindly provided by Sohyun Ahn (Ahn and Joyner, 2004, 2005). These mice express an inducible Cre recombinase (*CreER^T2^*) from the *Gli1* locus which is active in proliferative regions of the brain during development and in adults (Ahn and Joyner, 2005). *CreER^T2^* recombinase activity is activated on administration of tamoxifen (TM). *Gli1-CreER^T2^* mice were crossed with *Tau^mGFP^* mice (kindly provided by Silvia Arber) expressing a floxed myristoylated alanine-rich C-kinase substrate (MARCKS) protein fused to green fluorescent protein (GFP). *CreER^T2^* recombinase activity in *Gli1-CreER^T2^;Tau^mGFP^* mice leads to expression of membrane localized EGFP (Narboux-Nême et al., 2008; Dondzillo et al., 2010; Gangarossa et al., 2012).

### TM administration

*CreER^T2^* recombinase activity was induced by administration of TM in *Gli1-CreER^T2^;Tau^mGFP^*. A 62 mg/ml stock solution of TM (Sigma, catalog #T5648) was made fresh for each injection. TM powder was dissolved in a solution of 84% corn oil (Sigma)/16% ethanol prewarmed to 37°C. After vortexing for ∼2–3 min at room temperature (RT), stock TM solution was wrapped in foil and placed on a rotating mixer at 37°C for 3 h. Before each injection solution was examined for presence of air bubbles and/or precipitate. Mice were administered TM at a dose of 200 mg/kg via intraperitoneal injection. As a measure of determining whether TM administration was effective, the number of astrocytes in cerebral cortex and dorsal thalamus was qualitatively assessed (see Results). Animals were not included if little or no astrocyte labeling could be detected in these regions.

### Preparation of brain sections

Mice were killed by a lethal dose of fatal plus (Vortech Pharamaceuticals) through intraperitoneal injection. Once completely anesthetized, mice were intracardially perfused with a 2–3 ml of ice-cold saline [154 mm NaCl and 10 mm phosphate buffer (PB), pH 7.4] with heparin sulfate (10 U/ml). Following prefix saline rinse, mice were perfused with 20–30 ml of ice-cold formaldehyde solution prepared fresh from powdered paraformaldehyde (PFA; powder Electron Microscopy Sciences, catalog #19208) in 100 mm PB, pH7.4. The exact volume of fixative was determined by weight (1 ml/g) but was not <20 ml and not >30 ml. Following perfusions, brains were removed from the skull and cryoprotected in 10% sucrose, 0.1 M PB overnight at 4°C, then transferred to a solution of 30% sucrose, 0.1 M PB until they sank to the bottom of the tube (24–48 h).

### Immunofluorescent labeling of tissue sections

Frontal sections (30 μm) of mouse brain were generated using a freezing microtome and maintained in 100 mm PB until use. The composition of wash and block buffers were same as below for immunohistochemistry. All washes were performed at RT for 10 min. Briefly, after three washes sections were incubated in block buffer with additional 5% normal serum then transferred to block buffer with primary antibody overnight at 4°C. Multiplex immunofluorescent labeling was performed with a polyclonal rabbit antibody against GFP (Thermo Fisher, catalog #A-11122, RRID: AB_221569), a monoclonal antibody against NeuN (Millipore, catalog #MAB377, RRID: AB_2298772) and a guinea pig polyclonal antibody against doublecortin (DCX; Millipore, catalog #AB2253, RRID: AB_1586992). After three washes, tissue was placed in block buffer containing fluorescently conjugated secondary antibody and 4’,6-diamidino-2-phenylindole dihydrochloride (DAPI; 400 ng/ml, Invitrogen) for 1 h at RT. The following secondary antibodies were used for multiplex immunolabeling: goat anti-rabbit Alexa Fluor 488 (Thermo Fisher Scientific, catalog #A-11008, RRID: AB_143165), goat anti-mouse Alexa Fluor 647 (Thermo Fisher Scientific, catalog #A-21236, RRID: AB_2535805), and goat anti-guinea pig Alexa Fluor 594 (Thermo Fisher Scientific, catalog #A-11076, RRID: AB_141930). Finally, tissue was washed three times, mounted onto Superfrost Plus microscope slides (Thermo Fisher Scientific), and coverslipped using VWR VistaVision #1.5 cover glass (VWR Scientific) and ProLong Gold Antifade mountant (Thermo Fisher Scientific).

### Immunohistochemistry

Frontal sections (50 μm) of mouse brain were generated using a freezing microtome and maintained in 100 mm PB until use. All washes were done in 100 mm PB for 10 min at RT. Briefly, after three washes free floating sections were transferred to block buffer [5% normal serum (Vector Laboratories) and 0.3% Triton X-100 (Sigma) in 100 mm PB] supplemented with an additional 5% normal serum for 1 h at RT and then incubated overnight at 4°C in block buffer containing primary antibody. In order to visualize GFP marker expression in *Gli1-CreER^T2^* mice, we performed immunohistochemistry using a polyclonal rabbit antibody against GFP protein (Thermo Fisher, catalog #A-11122, RRID: AB_221569). After three washes, tissue was transferred to block buffer containing biotinylated secondary antibody for 1 h at RT, washed, then incubated in avidin-biotin complex (ABC; Vector Laboratories) in 100 mm PB for 1 h at RT. Finally, tissue was washed three times, and antibody labeling was visualized by reaction with the chromagen diaminobenzidine (DAB; Sigma). Sections were mounted onto Superfrost Plus microscope slides (Fisher Scientific), defatted in chloroform (Sigma), dehydrated through graded series of alcohol dilutions (50–100%), cleared in 100% xylene (Sigma) and finally coverslipped using Permount (Fisher Scientific) mounting media.

### Imaging

Tissue sections labeled by immunohistochemistry were imaged for axon reconstructions (see below) or by light microscopy using a Zeiss Axioimage M2 microscope with a Hamamatsu ORCA Flash4.OLT digital camera attached to a HP Z840 PC running Zen 2.3 (blue edition) software. Images of fluorescent immunolabeled sections were acquired using a Keyence BZ-9000 epifluorescent microscope and a Nikon 20×/0.75 Plan-Apo objective attached to a Dell precision T3610 PC running BZ-II Viewer software (v.2.1). For presentation, image files were imported into Adobe Photoshop (Adobe Systems) where linear adjustments to contrast and brightness and cropping was performed. Final figures were composed using Adobe Illustrator (Adobe Systems). Within a figure, all image panels were treated equally unless otherwise noted.

### Cell-type marker analysis

To analyze cell maturation post-TM administration, sections processed for multiplex immunofluorescent labeling for GFP and NeuN or GFP and DCX were counted under live epifluorescent imaging to determine cell numbers. Three sections from dorsal hippocampus from three separate animals were used for analysis. Cells were counted from the entire dentate gyrus of each section and averaged over all animals.

### Axon reconstructions and measurements

A total of 808 terminal boutons were reconstructed from 128 labeled mossy fiber axons from three animals at each time point using Neurolucida (MBF Bioscience) software using a Zeiss Axioscope 100 microscope (Carl Zeiss) equipped with motorized stage controls for the *x*-, *y*-, and *z*-axes and a color charge-coupled device (CCD) camera. Traces were generated at high magnification using a Zeiss Apochromat 100×, 1.4 numerical aperture, oil-immersion lens. Axon reconstructions were obtained from dorsal hippocampal sections and in stratum lucidum between the tip of the dorsal blade on the dentate gyrus and the beginning of the bend in Ammon’s horn of region CA3 (Fig. 2*D*,*E*). Measures of axon and MFB length, width, and volume were obtained during live tracing. Digitized axon traces were imported into Neuroexplorer (MBF Bioscience) for quantitative analysis. For quantification, MFBs were sorted into three categories, large complex boutons (MFBs; *n* = 409), “drumstick” or “lollipop-like” terminals (*n* = 83) and small swellings (*n* = 316; Acsády et al., 1998; Amaral and Levenex, 2007). MFBs were relatively large structures with diameters ranging from 2.5 to ∼3.5 μm that in the main were located in line with the main axon collateral or at the end of a short branch emanating from main collateral axon (Fig. 2*A*). Lollipop synapses were small in number and were categorized as a small circular swelling at the end of a collateral branch usually emanating at 90° from the main collateral axon. Swellings were categorized as relatively simple small enlargements of the main axon collateral that were usually no more than 1 μm in diameter and were always in line with main axon. While the significance of small swelling and lollipop terminals remains to be further investigated, our density analysis showed that the densities of both lollipop terminals and swellings were not significantly changed during maturation at all ages (Fig. 2*I*,*J*). We therefore focused these analyses on the large complex boutons (MFBs).

### EM

Tissue blocks containing hippocampus were cut with a vibratome (Leica) at 50–60 μm and sections were saved in the cold 100 mm PB. Sections were then processed for immunoperoxidase staining to visualize GFP-labeled components. Immunolabeled profiles were identified under light microscope in wet sections, areas showing dense labeled mossy fibers and their boutons were selected for EM embedding. The selected vibratome sections were washed in 100 mm PB solution, dehydrated in serial 50%, 70%, 90%, 95%, and 100% ethanol. Sections were further dehydrated in 100% acetone and flat embedded into Araldite. Embedded sections were polymerized in oven at 60°C for 48 h. The embedded sections were then examined in the light microscope 40× to identify immunoperoxidase-labeled GFP-positive mossy fibers and their boutons. CA3 region and partial dentate gyrus were carefully selected for EM sectioning. Serial thin sections from each sample were cut with an ultramicrotome (EMUC7, Leica), section thickness was 70 nm. Every 8–12 serial sections were collected to Formvar coated single slot copper grids. All the grids with serial sections were stored in order in the plastic grid box. For each sample, grids containing serial sections were counter stained with uranyl acetate and lead citrate for EM imaging.

For each sample, a range of 16–45 serial thin sections were examined in a Philips CM120 EM at 80 kV. GFP-labeled MFBs were identified and the low magnification images showing GFP labeled profiles and their associated synaptic components were taken in a serial order, images were captured with a 2000 × 2000 high-resolution CCD camera (Gatan Inc.). Serial EM images were saved using DigitalMicrograph software (Gatan Inc.), further processed in Adobe Photoshop CS by adjusting only the brightness and contrast.

### EM 3D reconstruction and analysis

To study the spatial relationship between GFP-labeled MFBs and their surrounding subcellular structures, we reconstructed serial EM images three dimensionally using RECONSTRUCT software (NIH). For each labeled MFB, a completed reconstructed profile, including GFP-labeled mossy fiber and MFBs, the postsynaptic dendritic components, and the associated non-labeled preexisting MFBs and astrocyte, was rotated and viewed from different angles to examine their spatial relationship. A total of 19 GFP-labeled MFBs and their surrounding structures were reconstructed with each time point having at least three reconstructed adult-born MFBs for analysis (Figs. 4, 5, 7). These reconstructed profiles were used for EM quantitative analysis. Two additional smaller newborn MFB at 4 weeks after injection at 3 months old (3M4W) were also reconstructed for comparison (Fig. 6).

To quantify proportion of spines targeted by labeled MFBs, TEs were identified in 3D reconstructions. Normalization of spine head counts was performed by dividing the number of spine heads contacted per labeled MFB by total number of thin serial sections used for reconstruction (*n* = 3 or 4 reconstructed MFBs at each time point). TEs were identified based on the 3D reconstructed GFP labeled boutons and their associated structures. The percentage of shared TE was then calculated. At each time point, 8–15 reconstructed TEs were identified.

For axonal bouton density analysis, before EM processing immunolabeled MFBs were identified in wet sections at each time point by light microscopy (*n* = 50–100 MFBs per section).

### Statistical analysis

Morphometric measures of areal and intra-axonal MFB densities between ages and post-TM intervals obtained from Neurolucida were statistically analyzed using two-way ANOVA followed by *post hoc* Student’s *t* tests. Values are reported as mean ± SEM. Relative changes in MFB size between ages and post-TM time points were made using frequency distribution plots and statistical significance was determined by the Kolmogorov–Smirnov test (Smirnov, 1948; Duan et al., 2007). Pair-wise comparisons in axon MFB density and TE spine head density at the ultrastructural level using EM were made using Student’s *t* tests. Values are presented as mean ± SD. In all cases, differences were considered significant with *p* < 0.05.

### Data availability

All data discussed in the manuscript will be made available on request.

## Results

### Adult-born neuron maturation in *Gli1-CreER^T2^;mGFP* mice

In order to investigate neurogenesis in adult hippocampus, we used a transgenic reporter mouse line *Gli1-CreER^T2^;Tau^mGFP^* (GliCreGFP), in which membrane bound enhanced GFP (mGFP) is conditionally expressed under control of the Tau promoter in neural stem cells following administration of the prodrug TM (Ahn and Joyner, 2005; Fig. 1). In *Gli1-CreER^T2^* mice, we observed labeling in neurons within adult hippocampus as well as astrocytes throughout brain including hippocampus, neocortex and dorsal thalamus (Fig. 1). Because astrocyte labeling seemed to occur regardless of age and/or time post-TM, we used this as an indication that TM administration was successful (see Materials and Methods). We examined MFB maturation by labeled newborn cells and their processes at different times post-TM injection in both young adult and aged animals (Fig. 1). Compared with other model systems which displayed limited utility in aging brains, TM administration in GliCreGFP mice efficiently labeled newborn GCs and their processes in young adult and aged brains (Fig. 1).

In general, the spatiotemporal pattern of newborn GC development in GliCreGFP mice was qualitatively similar to previous reports (Nicola et al., 2015; Wu et al., 2015; Toni and Schinder, 2016). In both adult and aged brain, labeled newborn GCs displayed a cell body contained within the GCL, a primary apical dendrite that branched near the junction of the molecular layer and continued to the hippocampal fissure and the presence of spines along dendrites (Fig. 1*A–F*). In addition, in both adult and aged brains, an axon was clearly visible emanating from the cell body and coursing through the CA3sl. These axons were contained within the mossy fiber bundle. At least three different morphologically distinct cell types were labeled post-TM injection. These included mature postmitotic GCs (Fig. 1*G*), astrocytes (Fig. 1*H*), and type 1 progenitors referred to as radial glial-like cells (RGLCs; Fig. 1*I*). In the dentate gyrus, the majority of labeled cells within the GCL were mature GCs or RGLCs. Very few astrocytes were observed within the GCL.

Confirmation of cell type was also performed using cellular marker analysis (Fig. 1*J–M*), which showed a characteristic change in composition of cell type from 4 W post-TM injection (wpi) to 16 wpi. At 4 wpi, newborn GCs appeared morphologically mature and expressed the mature neuronal marker NeuN (Fig. 1*K*). Although not all comparisons were statistically significant, the number of mature GCs increased at 8 wpi (Fig. 1*M*) and remained at this level by 16 wpi. There was a concomitant decrease in the number of cells co-immunolabeled with DCX, a marker of immature neurons, suggesting that newborn GCs were continuing to mature through 8 wpi in the GliCreGFP model (Fig. 1*J–M*).

### MFB maturation in *Gli1-CreER^T2^;mGFP* mice

To measure changes in MFB maturation, we performed a quantitative morphometric analysis along newborn mossy fiber axons within CA3 region at light microscopic level. These boutons were heterogenous in shape and size and could be grouped into three categories: large complex boutons (Fig. 2*A*), lollipop terminals (Fig. 2*B*), and small en passant swellings (Fig. 2*C*; for definitions of these categories, see Materials and Methods). Since the significance of the lollipop synapses and small swellings are not clear and our preliminary analysis indicates that these two categories do not show significant changes throughout maturation at various ages (Fig. 2*I*,*J*), we focused on large complex boutons.

Tissue sections processed for immunohistochemical labeling of GFP were used for population density MFB measures and axonal reconstructions using Neurolucida software (Fig. 2*D–E*). In order to assess the ability to form adult-born MFBs at various ages, we first estimated the overall population density of MFBs in stratum lucidum in 3-M-, 6-M-, 12-M-, and 18-M-old animals at 8 wpi (Fig. 2*F*). Bouton counts were made in 100-μm stretches of stratum lucidum. A total of 1162 MFBs were counted from between three and five animals at each time point. In order to control for differences in efficacy of TM administration, for each animal, the number of labeled GC bodies in the dentate was also counted and total MFB counts were divided by this number to normalized MFB measures between animals. Surprisingly, we found a peak increase in labeled MFBs within CA3 stratum lucidum at 6 M of age (Fig. 2*F*). A significantly larger number of surviving terminals were counted when injections were made at 6 M compared with 3 M (190% increase, *p* < 0.01) or 18 M (78% increase, *p* < 0.01). Increases in MFB terminal number, but to a lesser extent, were also observed post-TM injections in 12-M-old animals.

The same labeled tissues were used to perform single axon analyses. Axons were traced within a region from the medial border of CA3 through stratum lucidum as far as possible until a break in the axon was detected. Faulkner et al. (2008) previously showed in young adult animals that it takes eight weeks for an MFB to be morphologically mature. We therefore chose 4, 8, and 16 wpi to study MFB changes in presynaptic and postsynaptic structural elements during this time. Previous work suggested interbouton density was a characteristic feature of mature en passant MFBs (Amaral and Dent, 1981). We measured the MFB density (Fig. 2*G*) and interbouton distance (Fig. 2*H*) along single axons to access the changes of MFB formation on single axons during AHN. In young adult animals (3 M), there was a dramatic decrease in MFB density from 4 to 8 wpi (4 wpi, 32.7 × 10^−3^/μm, 8 wpi, 13.5 × 10^−3^/μm, *p* < 0.005; Fig. 2*G*). The decrease was almost threefold at 16 wpi (4 wpi, 32.7 × 10^−3^/μm, 16 wpi, 11.6 × 10^−3^/μm, *p* < 0.005). Consistent with this observation, the interbouton distance increased from 4 to 8 wpi in young adult mice and reached ∼75 μm at 16 wpi (4 wpi, 43.9 μm, 16 wpi 74.6 μm, *p* < 0.05; Fig. 2*H*).

Previous EM analysis of newborn MFB development in young adult brain found MFBs can reach a mature perimeter size at 4W, but it takes approximately 8W for the newborn MFBs to have a mature synapse density (Faulkner et al., 2008). To better quantitatively understand the size distribution of MFBs during AHN, we measured the diameter of each newborn MFB at 4, 8, and 16 wpi and plotted the accumulative distributions of the MFB diameters (Fig. 3*A–C*). In young adult brain (3 M), we observed a small increase in mossy fiber diameter from 4 to 8 wpi, but no further significant changes were observed between 8 and 16 wpi (Fig. 3*A*). This quantitative analysis is consistent with prior observations that in young adult brain, newly formed MFBs reach mature size 4W after they are born.

### Age-related changes in MFB maturation during AHN

Considerable evidence suggests that adult neurogenesis is severely reduced in aging brains. However, the integration of new neurons into existing hippocampal circuitry and the functional plasticity to hippocampal circuitry in aged brain have not been addressed. To determine whether newborn neurons in aged brain underwent a maturation process similar to young adult we measured MFB terminal densities along newborn mossy fiber axons (Fig. 2*G*). At 4 wpi, the MFB densities were significantly lower in middle age (12 M) and aged (18 M) mice compared with young (3 M) adults (3M4 wpi, 32.7 × 10^−3^/μm, 12M4 wpi, 8.7 × 10^−3^/μm, 18M4 wpi, 10.9 × 10^−3^/μm, *p* < 0.005). In sharp contrast to young adult animals, the MFB densities were gradually increased from 4 to 16 wpi in 12 M and 18-M-old mice. While such an increase in 12 M middle age mice was not dramatic, there was nearly a 2.5-fold increase in MFB density in 18 M aged mice from 4 to 16 wpi (18M4 wpi, 10.9 × 10^−3^/μm, 18M16 wpi, 23.3 × 10^−3^/μm, *p* < 0.01). As a consequence, at 16 wpi, MFB density was twofold higher in 18 M aged mice compared with 3 M young adults (3M16 wpi, 11.6 × 10^−3^/μm, 18M16 wpi, 23.3 × 10^−3^/μm, *p* < 0.01). Interbouton densities also showed differences between young adult and aged mice (Fig. 2*H*). While interbouton density increased with maturation in young adult animals (3 M), interbouton distances decreased in middle age (12 M) and aged (18 M) mice. In line with MFB density analysis, interbouton spacing was significantly lower in 18 M animals after 16 wpi (18M4 wpi, 110.4 μm, 18M16 wpi, 48.2 μm, *p* < 0.05; Fig. 2*H*). Together, these observations indicate that there are significant differences in the dynamics of new-born MFB development in young and aged brain: in young adult brain, numerous MFBs are formed at 4 wpi, but many of them disappear later at 8 and 16 wpi. In aged brain, however, fewer form initially at 4 wpi, but MFBs are continuously added and their density can be even higher than young brain at 16 wpi.

A different pattern of MFB terminal size maturation was also observed. In aged mice (18 M), MFB diameter decreased over time (Fig. 3*B*,*D*,*E*). At 4 wpi, the population of MFB were on average relatively large. From 4 to 16 wpi, however, their average size decreased. When MFB sizes were compared between ages at 4 wpi it is apparent that middle age (12 M) and aged mice (18 M) have a significantly higher population of large MFBs compared with young adults (3 M; *p* < 0.005, Kolmogorov–Smirnov test for distribution difference; Fig. 3*C*). The trajectory of MFB size changes during maturation are strikingly different between age groups (Fig. 3*D*,*E*): while the average MFB size in 3-M-old mice increases from 4 to 16 wpi, the average size in 18-M-old mice decreases. What is also interesting is that by 16 wpi the average MFB size is similar in adult (3 M) and aged (18 M) animals (Fig. 3*E*). These data indicate that in young adult brain a pruning mechanism refines MFB development, while in aged brain a progressive accumulation of MFBs from larger to smaller sized overall occurs during maturation.

### Ultrastructural analysis of MFB integration in young adult and aged brain

Apparent differences in MFB number and size during maturation between young adult and aged animals suggests the developmental plasticity of the integration of these newborn MFBs is altered with aging. In order to investigate in high resolution morphologic changes in MFB maturation, we performed quantitative immuno-EM at 4, 8, and 16 wpi in young adult (3 M) and aged (18 M) GliCreGFP transgenic mice. We used a serial immuno-EM approach to reconstruct and quantify the development of newborn MFBs labeled in the GliCreGFP mice focusing on larger MFBs that terminate with TEs on the proximal region of CA3 pyramidal neurons to concentrate our analysis on the more developed newborn MFBs at each time point.

A total of 19 labeled large MFBs at 4, 8, and 16 wpi from 3 M young adult and 18 M aged brains were reconstructed and analyzed. Three MFBs at each time point were analyzed except at 3M4 wpi, where four MFBs were analyzed. Each MFB was reconstructed from on average 32 serial thin sections (ranging from 16 to 45 sections). Consistent with observations at the light microscopic level, in EM preparations we found that MFB densities were significantly reduced from 4 to 16 wpi in 3 M mice, while densities gradually increased, but were not statistically different, along the same timeline in 18 M mice (3 M: 32 × 10^−3^/μm, 18W: 12 × 10^−3^/μm, *p* < 0.05; *n* = 50–100 boutons at each time point; Fig. 4*A*).

It has been previously reported that in 2-M-old young adult mice adult-born MFBs initial form synaptic contacts on dendritic shafts of CA3 pyramidal cells by two weeks (Faulkner et al., 2008). By four weeks, many of these new boutons reached a mature size and contained a mature number of invading TEs. The number of synaptic contacts within each bouton continued to increase until eight weeks when the new MFBs reach morphologic maturity and remain stable through at least 16 weeks (Faulkner et al., 2008). Consistent with this timeline, we found that MFBs at 4 wpi in 3-M-old mice had already exhibited invaginating TEs with multiple spine heads on each TE. To further analyze the maturation of MFBs from 4 to 16 wpi, we quantified the development of spine heads on TEs (Fig. 4*B*). The normalized spine head counts (see Materials and Methods) in the 4 wpi samples from 3-M-old mice were low, but doubled by 8 wpi and remained the same at 16 wpi, indicating that the postsynaptic components of adult-born MFBs in young adult brain also undergo a temporal maturation process from four to eight weeks (3M4 wpi: 8 × 10^−2^ normalized spines/MFB, 3M8 wpi: 18 × 10^−2^ normalized spines/MFB, *p* < 0.05; *n* = 3–4 reconstructed boutons at each time point). By contrast, the samples from 18 M aged brain showed a very different pattern: normalized spine head counts remained stable from 4 wpi to 16 wpi (18M4 wpi: 20.6 × 10^−2^ normalized spines/MFB, 18M8 wpi: 20.3 × 10^−2^ normalized spines/MFB) and at numbers similar to mature 3 M, 16 wpi animals (3M16 wpi: 18 × 10^−2^, 18M16 wpi: 21 × 10^−2^ normalized spines/MFB, *p* = 0.19; Fig. 4*B*). This surprising finding suggests that newborn MFBs in aged animals are complex from earliest stages of maturation and remain so over time.

### Sharing of newly formed MFBs with old MFBs in young adult brain is common even after the new MFBs are morphologically mature

Previous studies have suggested that newborn MFBs in adult brain compete with preexisting MFB for terminal space on postsynaptic TEs (Toni et al., 2008; Toni and Schinder, 2016). That is, the labeled newborn MFBs are shown to “share” a TE with an unlabeled more mature MFB. It is suggested that over time the newborn MFBs progressively occupy more of the TE and eventually outcompete for sole occupancy of the TE. In order to understand the dynamic of sharing during the maturation of adult-born MFBs, we focused our analysis on whether and how each reconstructed MFB from various timepoints exhibited sharing with neighboring mature MFBs.

In 3 M young adult samples, we clearly saw sharing at four weeks, the earliest stage analyzed. As shown in Figure 5*A–C*, a representative four-week-old MFB was just starting to invade an old mature MFB by pushing away the surrounding astrocytic processes and sharing a TE with the old MFB. More robust sharing was also observed in eight-week-old MFBs (Fig. 5*D–G*). Unexpectedly, we also observed sharing in the 16-week-old MFBs (Fig. 5*H–K*), although by this time these MFBs should be fully mature. When we quantified the fraction of each shared TE that was in contact with a newborn MFB we observed 39% at 4 wpi, and this remained fairly constant through 16 wpi, 44% of shared TE per MFB (Fig. 4*C*; *n* = 8–15 reconstructed TEs for each time point) These results suggest that sharing of newborn MFBs with old MFBs is very common in young adult brain, even after the MFBs are morphologically fully mature.

The four 4 wpi MFBs from 3 M young adult animal were classified as stage 3a based on previously published classifications (Faulkner et al., 2008). In order to understand more the sharing dynamic, we reconstructed an additional pair of stage 2 small MFBs at 4 wpi that were on a segment of newborn mossy fiber 40 μm apart (Fig. 6). This segment of mossy fiber seemed to have contacts with dendritic shaft and spine heads from a postsynaptic CA3 pyramidal cell. A clear sharing with a spine head that was mostly encircled by a mature MFB was observed in one of the new MFB. Thus, based on previous results (Faulkner et al., 2008) and the current study, we conclude that in young adult brain, the integration of MFBs into existing mature neural circuit is a highly dynamic process involving both *de novo* formation of new synaptic contacts with postsynaptic dendritic shaft and takeover of preexisting mature MFB by invasion and sharing.

### Sharing and replacing preexisting old MFB as a major mechanism for integration of newborn MFBs in aged brain

Sharing also occurred in the samples from 18 M aged brain. Quantification of the percentage of each shared TE that was in contact with a newborn MFB (Fig. 4*C*) showed that 39% was observed at 4 wpi, but this dropped dramatically to only 11% at 8 wpi, and no sharing was observed from 16 wpi samples (Fig. 4*C*). An example of sharing at 8 wpi is shown in Figure 7*A–C*, in which the invading new MFB almost replaced the old MFB. At 16 wpi (Fig. 7*D–I*), the MFB was mature and was morphologically no different from the neighboring preexisting MFBs. In addition, no evidence of *de novo* synapse formation was observed in all the 18-M-old samples we analyzed. It is important to note that in this current study an average 32 serial thin sections were used for reconstructing MFBs. Each thin section for EM was 70 nm. The thickness analyzed for each MFB is therefore at least 2 μm which is sufficient enough to cover large portion of MFB and its associated TEs. This explains why we were able to detect sharing in many MFBs at various timepoints. Thus, the fact that we observed no *de novo* synapse formation in all samples and no sharing at 16 wpi sample in 18 M aged brain strongly suggests a limited integration capacity of newborn MFBs in aged brain.

The lack of sharing in 16 wpi and no evidence of *de novo* synaptic formation in 18 M aged brain were surprising. However, these findings were consistent with the finding that postsynaptic TEs with spine heads were morphologically mature throughout the 4 to 16 wpi period in 18-M-old brains (Fig. 4*B*) and suggests the major mechanism for newborn MFB integration in aged brain is through replacement of preexisting MFBs. Unlike young adult brain, sharing in the aged brain is relatively transient. Once mature, new MFBs completely replace old MFBs without further sharing in later stage.

## Discussion

### Integration of newborn MFBs in aged brain exhibits features distinct from those in young adult brain or in early developing hippocampus

One hallmark feature of the aging hippocampus is an apparent loss of hippocampal adult neurogenesis (Kuhn et al., 1996; Klempin and Kempermann, 2007; Morgenstern et al., 2008; Jinno, 2011, 2016; Kempermann, 2011; Lee et al., 2012; Kempermann et al., 2015). Rates of cellular proliferation are dramatically reduced in adult brain relative to early developing brain and these rates continue to decline throughout old age (Rao et al., 2006; Olariu et al., 2007; Hattiangady and Shetty, 2008). Similar reductions in the number of mature adult-born neurons have also been described raising the possibility that the ability of newborn neurons to functionally integrate into the mature preexisting circuitry of the hippocampus is compromised with aging (Kuhn et al., 1996; Bondolfi et al., 2004; Heine et al., 2004). Here, we show that, in contrast to that notion, the axonal output of newborn GCs in aged hippocampus are fully capable of functional integration onto the mature circuitry. What is more the morphometric features of these newborn MFBs after maturation in aged brain resemble those found in young adult brains including average MFB size, density, and interbouton distance. On the other hand, our analysis also reveals that the integration dynamics of newborn MFBs in young adult and aged hippocampus are distinctively different.

In young adult hippocampus, a large population of relatively smaller MFBs are generated at early stage of integration, but they are gradually pruned to reach a smaller population of mature-sized boutons at 8 weeks and remain constant at 16 weeks. The postsynaptic TEs also undergo maturation from four to eight weeks by increasing the number of spine heads and *de novo* synaptic formation is common (Faulkner et al., 2008). Interestingly, sharing is prevalent throughout the entire integration process and persists even at 16 weeks. Similar to the poly-innervation of muscle fiber in neuromuscular junction (NMJ) during embryonic development, sharing is an active competitive process between newly formed MFB and preexisting mature MFB. The new MFB can completely replace the mature one if the former outcompetes the latter. In the young adult brain, the percentage of sharing continuously increases and is the highest at 16 weeks, suggesting that it might take a long time if replacement does occur. Thus, the integration of newborn MFBs is a highly dynamic process in young adult brain, involving *de novo* synaptic formation and vigorous competition with preexisting MFBs. The loss (so called pruning) of newborn MFBs over time in young adults suggests preexisting MFBs may often be able to outcompete new ones. Overall, this process is more likely to end with the addition of synaptic inputs rather than their replacement.

In aged hippocampus, a small population of larger than average mature boutons are required for the early stage of MFB integration. Additional MFBs are added over time and the average overall size becomes comparable to that of mature boutons at 16 weeks in a 3 M young adult. What is also unique is that from four to 16 weeks the morphology of postsynaptic TEs remains mature and no evidence of *de novo* synaptic formation is observed despite rigorous attempts to detect it. Sharing of the new MFB with old MFB is more common at 4 weeks but is significantly reduced at 8 weeks and totally disappears at 16 weeks. The fact that no sharing can be observed at 16 weeks suggests that newly formed MFBs in aged brain can always outcompete and replace preexisting MFBs. This suggests some deficiency in aged MFBs in their ability to be competitive for synaptic targets. Thus, the integration dynamics for newborn MFB in aged brain appear relatively limited and synaptic replacement might be their primary mechanism for integration. These observations could have important implications for the functional role of adult neurogenesis in aged brain.

Previous studies have shown that the maturation time course of newborn MFBs in adult hippocampus is longer than in early postnatal weeks when the hippocampus is formed. However, the morphologic features of MFBs born in adult brain are in general consistent with features previously described in rodent during early development but with some notable differences (Blackstad and Kjaerheim, 1961; Stirling and Bliss, 1978; Amaral and Dent, 1981; Amaral et al., 1990; Amaral and Levenex, 2007). The MFBs are large irregularly shaped structures that are found to be either in direct line with the mossy fiber axon or are at the end of short collateral branch. Previous EM reconstruction studies on early postnatal hippocampus shows that over time the complexity of the MFB synaptic complexes become more intricate with increased numbers of spines, PSDs, and mitochondria. The large irregular MFBs have been consistently described as having diameters between 3 and 6 μm (Blackstad and Kjaerheim, 1961; Stirling and Bliss, 1978; Amaral et al., 1990). In line with these observations we found that 60% of adult-born MFBs in young adult and aged animals were between 2 and 4 μm in diameter after 16 weeks (Fig. 3*A*,*B*). Interbouton distance in newborn MFBs in young adult brain is also consistent with prior observations in early development. Previous EM studies in adult rats have described MFB densities of one to two terminals per 100 μm (Stirling and Bliss, 1978; Amaral et al., 1990) which is in line with the density of 1.2 MFB per 100 μm reported here (Fig. 2*G*). However, it is interesting to note that in aged brain MFB densities continue to increase and reach more than twice the density of newborn MFBs in young adult brain at 16 weeks (2.5 MFB per 100 μm). Notably, while density of terminals per axon is greater when comparing 18-M- to 3-M-old animals, the overall population density of MFBs is not significant different (Fig. 2*F*). This suggests a reduced axonal density which would be consistent with the reported reduction in neurogenesis in aged brain. It is possible that the increased density in axonal MFBs is a compensatory measure in response to reduced neurogenesis.

As stated above, aging reduces the propensity for neurogenesis in adult hippocampus. This has been documented in multiple species including human and non-human primate brain (Ngwenya et al., 2015; Boldrini et al., 2018; Sorrells et al., 2018; Moreno-Jiménez et al., 2019), suggesting a fundamental age-related decline in functional plasticity associated with adult neurogenesis. However, it is not clear whether this decline is limited to neuron number or also impacts their ability for functional integration. Trinchero et al. (2017) have shown that middle-aged mice (5–8 M) have delayed dendritic and spine maturation on newborn GCs but that morphologic features are eventually similar to GCs from 2-M-old animals. What is more, voluntary exercise restores developmental rates to young adult suggesting mechanisms of activity-dependent plasticity are intact. Similarly, we found that the rate of maturation of adult-born MFBs was altered in aged animals but that ultimately many morphometric features were similar. So, whereas in young adult brain the final density of MFBs is arrived at through progressive removal of contacts, in aged brain densities increase over time to arrive at a similar overall bouton density. This suggests that mature features of GC morphology may be intrinsically determined, while cellular environment (i.e., young vs aging brain) may influence the dynamics of maturation.

### Replacement of preexisting MFB with newborn MFB as a mechanism of circuit integration during adult neurogenesis

One of the most intriguing observations in the current study is the manner in which some adult-born MFBs form new contacts through a process of synaptic replacement. This process occurs through the progressive invasion and replacement of an existing, mature MFB, with a newly formed terminal. An obligatory intermediary step in this process thus involves the dual innervation of a single TE and its spine heads by two independent MFBs (newborn and preexisting mature boutons) which would seem to counter prior studies on MFB morphology in adult rodents where a consistent description has been the innervation of TEs by a single MFB (Blackstad and Kjaerheim, 1961; Amaral and Dent, 1981; Amaral and Levenex, 2007). However, full reconstructions of TEs at the EM level did reveal that a small subset can be innervated by two (or more) MFBs (Chicurel and Harris 1992). In those studies, it was unclear whether or not these MFBs were all established mature terminals or some were adult-born, but more recent studies have confirmed that adult-born GCs can form transient multi-innervations of TEs during maturation (Toni et al., 2008; Toni and Schinder, 2016). In addition, a recent serial block face EM reconstruction analysis in mice of MFB maturation during early development found a transient occurrence of postsynaptic TEs contacted by multiple MFBs before full maturation (Wilke et al., 2013). Collectively, these observations imply that, like synaptic maturation at the NMJ, during early postnatal formation of hippocampus, developing TEs can initially be innervated by multiple MFBs, which are refined to a single MFB-TE relationship at maturity (Buffelli et al., 2003). Whether a neuronal activity-dependent mechanism is responsible for pruning of inputs, as is the case at the NMJ, needs to be determined. Direct evidence supporting neuronal activity-dependent competitive integration of adult-born MFBs is mixed and limited by a lack of studies addressing competition at the single bouton level (Yasuda et al., 2011; Lopez et al., 2012). Whatever the cellular mechanisms, it is clear that in young adult brain sharing remains prevalent, while in aged brain newborn MFBs seem less likely to form *de novo* synaptic contacts by inducing brand new TEs from very old postsynaptic pyramidal cells. Rather, newborn MFBs in aged brain must be large and exhibit a strong competitive edge to replace preexisting old MFBs.

That adult-born MFBs can both add and replace existing contacts on presumably more mature terminals is a particularly interesting observation which could have important implications for hippocampal network function. While the role of AHN in hippocampal function is not known it has been suggested that hippocampal neurogenesis leads to degradation or forgetting of memories during infancy and in young adult (Akers et al., 2014). The competition and replacement between newborn and old MFBs during AHN might provide a mechanistic explanation. More specifically, it has been suggested that AHN plays a role in discrimination of contextual differences in episodic memory formation (Aimone et al., 2011; Sahay et al., 2011a,b; Johnston et al., 2016). Blocking neurogenesis in adult brain inhibited a critical component of episodic memory, pattern separation (Nakashiba et al., 2012). In contrast, blocking activity of existing mature GCs while leaving newborn GCs intact did not affect pattern separation. This suggests AHN underlies a vital component of episodic memory. Intuitively, the addition of novel “nodes” to the hippocampal network and the replacement or erosion of existing nodes could be a cellular substrate regulating such discriminatory function. In this regard, the absence of observable *de novo* synapse formation in aged brain could be relevant to declining hippocampal dependent hippocampal function with age (Barnes, 1994; Shen et al., 1997; Rosenzweig and Barnes, 2003; Chawla and Barnes, 2007; Lister and Barnes, 2009; Burger, 2010; Samson and Barnes, 2013; Villanueva-Castillo et al., 2017).

## Synthesis

Reviewing Editor: Chay Kuo, Duke University Decisions are customarily a result of the Reviewing Editor and the peer reviewers coming together and discussing their recommendations until a consensus is reached. When revisions are invited, a fact-based synthesis statement explaining their decision and outlining what is needed to prepare a revision will be listed below. The following reviewer(s) agreed to reveal their identity: Shaoyu Ge.

Reviewer 1

This manuscript using confocal and EM methods briefly analyzed the axonal integration of newborn neurons in the adult and aged hippocampus. In briefly, in 3, 12- and 18-months old mice, they used Gli1-CreERT2 mice to birthdate and mark new neurons. Then they used confocal and EM methods to reconstruct the terminals and boutons of new neurons at different time after TM induction in the CA3. The work is interesting and can be an addition to the current known knowledge. I have the following comments to be addressed before a publication.

1) The whole manuscript suffers from shortage of statistic numbers. What are the animal numbers? What kind of statics have been done?

2) In figure 1, the quantification of M should be better presented. A column plot should be implemented so that we could have an idea of numbers.

3) Again, in the figures 2-3, the authors should provide details for animal information.

4) Figure 4 should be matched with related images in Figures 5-7. This comes down to the concern again. It will be necessary to know: for example in figure 7, “the authors concluded electron micrographs and 3D reconstructions demonstrating sharing of postsynaptic thorny excrescences by newborn and mature MFBs in 18M adult mice at 8W post TM but not at 16W post TM” , how many reconstructed images did the authors observe this phenomenon? How many animals? This conclusion needs to be well supported by the statistical data instead of a single reconstructed image. This applies to the figures 5 and 6 as well.

Reviewer 2

This is an interesting study that examines mossy fiber terminals of adultborn GCs in the adult and aging hippocampus.

Comments

-Please provide numbers of boutons AND animals used for each timepoint and for all experiments. A concern is that the measures maybe skewed if obtained from too few mice per timepoint.

-Variations in bouton density maybe due to numbers of RGLs labeled? How can the authors address this issue?

-The authors argue: “While this surprising observation might be confounded by TM injection variability, the relatively small standard error in each age group does support the notion that more mature MFBs are formed between 6M to 12M of age than 3M old”.

I do not agree.

(i) There does not appear to be a statistical diff btw 12 mos and 3 mos old mice.

(ii) How do the authors control for TAM variability and equivalence of labeling RGLs across ages?

-Please show scatter plots

p. 16

-The evidence for de novo synapse formation, unlike that for TE sharing, is too scant and hence not convincing.

## Author Response

We would like to thank the reviewers and editor for their positive feedback and comments regarding our submission. We are delighted the reviews were favorable and feel the comments have significantly improved the manuscript.

With regard to the species studied we have explicitly made reference to the fact that it is mouse in the abstract and will ensure the submission form also refers to mouse.

A point by point rebuttal to the reviews follows:

Point-to-Point Responses

Reviewer #1:

We would like to thank the reviewer for commenting that our studies are “interesting and can be an addition to the current known knowledge.”.

(1) The whole manuscript suffers from shortage of statistic numbers. What are the animal numbers? What kind of statics have been done?

We agree and have stated the animal numbers and “n” throughout the manuscript including in figure legends. In our original submission we stated in the methods section what statistics were used for each analysis. We have reiterated this throughout the manuscript.

(2) In figure 1, the quantification of M should be better presented. A column plot should be implemented so that we could have an idea of numbers.

We have changed the graph used and added stats where appropriate.

(3) Again, in the figures 2-3, the authors should provide details for animal information.

Animal numbers and “n” have been explicitly stated.

(4) Figure 4 should be matched with related images in Figures 5-7. This comes down to the concern again. It will be necessary to know: for example in figure 7, “the authors concluded electron micrographs and 3D reconstructions demonstrating sharing of postsynaptic thorny excrescences by newborn and mature MFBs in 18M adult mice at 8W post TM but not at 16W post TM” , how many reconstructed images did the authors observe this phenomenon? How many animals? This conclusion needs to be well supported by the statistical data instead of a single reconstructed image. This applies to the figures 5 and 6 as well.

We appreciate the reviewer’s comment and apologize for not clearly point this out. The figures in Figure 5-7 are representative figures. We have clarified the number of reconstructions etc. in the methods, results and figure legends. In addition, we have identified number of reconstructions and animal numbers in Figures 5-7 in order to better link them with Figure 4.

Reviewer #2:

We would like to thank this reviewer for commenting that our manuscript “is an interesting study”.

(1) Please provide numbers of boutons AND animals used for each timepoint and for all experiments. A concern is that the measures maybe skewed if obtained from too few mice per timepoint.

Throughout the manuscript we have explicitly stated the numbers for animals and terminals measured.

(2) Variations in bouton density maybe due to numbers of RGLs labeled? How can the authors address this issue?

The authors argue: “While this surprising observation might be confounded by TM injection variability, the relatively small standard error in each age group does support the notion that more mature MFBs are formed between 6M to 12M of age than 3M old”.

I do not agree.

(i) There does not appear to be a statistical diff btw 12 mos and 3 mos old mice. ?

The concern is reasonable. We have therefore altered the discussion in this paragraph to explain clearly the methods and results, as well as to tone down the interpretation. Since these observations do not affect our major conclusion, we feel there’s no need to remove Figure 2F.

(ii) How do the authors control for TAM variability and equivalence of labeling RGLs across ages

We gauged variability in TM administration by assessing the number of astrocytes labelled in cerebral cortex and dorsal thalamus. This has been stated in the text, “Results”, paragraph 1.

(3) Please show scatter plots

p. 16

It’s not clear to which figure the reviewer is referring as page 16 of the submitted manuscript contained no figures. However, Figure 1M was changed to a bar plot which may be the figure the reviewer is referring to.

(4)-The evidence for de novo synapse formation, unlike that for TE sharing, is too scant and hence not convincing.

We understand the evidence within the current manuscript is scant regarding de novo synapse formation. We did not present it for young adult animals because our previous work is convincing regarding this point (Faulkner et al. 2008). No significant evidence regarding de novo synapse formation was found in aged animals, in spite of an extensive effort to find some. We therefore think discussion of this idea is important. However, we have modified the discussion throughout to temper the conclusions regarding lack of de novo synaptogenesis.
